# Exhaled breath analysis with the use of an electronic nose to predict response to immune checkpoint inhibitors in patients with metastatic melanoma: melaNose trial

**DOI:** 10.3389/fimmu.2025.1564463

**Published:** 2025-04-03

**Authors:** Brigit van Dijk, Ivonne J. H. Schoenaker, Astrid A. M. van der Veldt, Jan Willem B. de Groot

**Affiliations:** ^1^ Department of Medical Oncology, Erasmus Medical Center Medical Center (MC), Rotterdam, Netherlands; ^2^ Isala Oncology Center, Isala, Zwolle, Netherlands; ^3^ Department of Radiology and Nuclear Medicine, Erasmus Medical Center (MC), Rotterdam, Netherlands

**Keywords:** melanoma, immune checkpoint inhibitor (ICI), eNose, clinical benefit prediction, exhaled breath analysis, volatile organic compound (VOC)

## Abstract

**Introduction:**

Immune checkpoint inhibitors (ICIs) have significantly improved the overall survival for patients with different solid tumors. However, there is an urgent need for predictive biomarkers to identify patients with metastatic melanoma who do not benefit from treatment with ICIs, to prevent unnecessary immune related adverse events (irAEs). Electronic noses (eNoses) showed promising results in the detection of cancer as well as the prediction of response outcome in patients with cancer. In this feasibility study, we aimed to investigate whether the breath pattern measured using eNose can be used as a simple biomarker to predict clinical benefit to first-line treatment with ICIs in patients with metastatic melanoma.

**Methods:**

In this prospective, observational single-center feasibility study, patients with metastatic melanoma performed a breath test using Aeonose™ before start of first-line treatment with ICIs. The detected exhaled breath pattern of volatile organic compounds (VOC) was used for machine learning in a training set to develop a model to identify patients who do not benefit from treatment with ICIs. Lack of clinical benefit was defined as progressive disease according to best tumor response using RECIST v1.1. Primary outcome measures were sensitivity, specificity and accuracy.

**Results:**

The eNose showed a distinct breath pattern between patients with and without clinical benefit from ICIs. To identify patients who do not benefit from first-line ICIs treatment, breath pattern analysis using the eNose resulted in a sensitivity of 88%, specificity of 79%, and accuracy of 85%.

**Conclusion:**

Exhaled breath analysis using eNose can identify patients with metastatic melanoma who will not benefit from first-line treatment with ICIs and guide treatment strategies. When validated in an external cohort, eNose could be a useful tool to select these patients for alternative treatment strategies in clinical practice.

## Introduction

Immune checkpoint inhibitors (ICIs) have significantly improved the overall survival for patients with different solid tumors (1). In patients with metastatic melanoma, the ten-year overall survival rates improved up to 43% after combination therapy with anti-programmed death (PD-1) and anti-cytotoxic T lymphocytes-associated antigen 4 (CTLA-4) directed antibodies (2). However, a substantial number of patients do not benefit from treatment with ICIs. Furthermore, ICIs are associated with severe, irreversible and potentially life-threatening immune related adverse events (irAEs) (3). Therefore, there is an urgent need for biomarkers that can early predict outcome after treatment with ICIs.

In patients with metastatic melanoma, predictive biomarkers could individualize the treatment with ICIs and select patients for different treatment strategies, including escalation from monotherapy to combination therapy or switching to targeted therapy. Predictive biomarkers could be useful to identify patients who will have clinical benefit from treatment with ICIs and, consequently, prevent unnecessary irAEs in patients who will likely not benefit. To date, however, reliable biomarkers to predict the efficacy of ICIs in patients with metastatic melanoma are lacking.

In patients with metastatic melanoma who are treated with ICIs, metabolic processes are activated as a result of tumor response to ICIs or disease progression. In tissue, metabolic processes produce volatile products that are released into the blood circulation and, once these reach the lungs, are passed on to the respiratory tract (4). Exhaled breath is mainly composed of inorganic compounds, such as nitrogen, oxygen, carbon dioxide, water vapor and inert gases. In addition, exhaled breath contains thousands of VOCs, which reflect pathological processes and generate unique patterns as a result of inflammation, oxidative stress or carcinogenesis (5).

From the exhaled breath, a breath pattern can be analyzed using an electronic nose (eNose). To analyze gaseous samples, these devices use pattern recognition, whereas traditional methodologies, including gas chromatography and mass spectrometry (GC-MS), are used to identify specific molecules in exhaled breath instead of a unique composite breath signal (6). In previous studies, eNoses were used to detect different diseases such as multiple sclerosis, inflammatory bowel disease, chronic kidney disease and solid tumors (7). eNoses are non-invasive and portable devices, designed to replicate human olfaction with the aim to capture a distinctive breath pattern and are promising for disease detection and even prediction of outcome after treatment. In oncology, eNoses showed promising results for the detection of colorectal cancer (8) and lung cancer (9–13), but also the differentiation between melanomatous lesions and benign skin lesions (14). In addition, a proof of concept study showed that an eNose can predict patients with advanced non-small-cell lung cancer (NSCLC) who will have an objective tumor response to treatment with anti-PD-1 antibodies (15). Since the number of patients who are treated with ICIs for solid tumors is increasing (16), the potential broader application of an eNose to predict outcome of treatment with ICIs seems promising.

In the current feasibility study, we aimed to investigate whether an eNose can be used as a simple and early biomarker to predict clinical benefit to first-line treatment with ICIs in patients with irresectable and metastatic melanoma. Early identification of patients who do not benefit from ICIs is needed since this could guide clinicians to start alternative, potentially more effective treatment strategies and prevent irAEs in patients who will not benefit from ICIs.

## Methods

### Study design

In this prospective, observational single-center feasibility study, we evaluated the predictive performance of the eNose in patients with advanced melanoma who were treated with first-line ICIs, either anti-PD1 monotherapy (nivolumab or pembrolizumab) or combination of anti-CTLA4 (ipilimumab) plus nivolumab. The study was performed in the Isala Oncology Center, Zwolle, the Netherlands. The Medical Ethics Review Committee (METC) of Isala has declared that the study protocol is not considered subject to the Medical Research Involving Human Subjects Act (WMO) in compliance with Dutch regulations. Informed consent was obtained from all participants before inclusion in the study.

### Study population

Patients were eligible when they were over the age of 18 years and planned to start with first-line treatment with anti-PD1 based treatment (nivolumab, pembrolizumab or ipilimumab plus nivolumab) for irresectable stage IIIC or stage IV cutaneous melanoma.

Previous systemic treatment was only allowed in the (neo)adjuvant setting. All included patients were treatment naïve for the treatment of advanced melanoma and received first-line systemic treatment with ICIs after the breath test.

Patients with secondary malignancies were excluded, except for completely resected basal cell and squamous cell skin cancers, any completely resected carcinoma *in situ* and malignancies that had been treated with curative intent at least two years before inclusion. Patients who were deemed physically incapable of performing the breath analysis were not included.

### Clinical data collection

Each patient was assigned an anonymous study number. Clinical characteristics were collected and included patient characteristics (age, gender, performance status), disease characteristics (primary melanoma, metastatic sites, lactate dehydrogenase (LDH) at baseline and treatment characteristics (administered ICIs, treatment duration and total number of cycles). In addition, potential exogenous and endogenous patient-related factors that could influence the VOC composition were collected. Exogenous factors included smoking, medication, alcohol, specific diet or fasting time, use of vitamins or herbal supplements (17). Endogenous factors included body mass index (BMI) and specific comorbidities, such as hypertension, diabetes mellitus, myocardial infarction, heart failure, asthma, chronic obstructive pulmonary disease (COPD), kidney failure, thyroid dysfunction, dementia, Parkinson’s disease, cerebrovascular accident (CVA) or infections (17).

Tumor response to treatment with ICIs was prospectively evaluated using a diagnostic computed tomography (CT) and in case of brain metastasis, MRI was also required for response evaluation. A low-dose CT of an acquired ^18^FDG-PET/CT was allowed if sufficient target lesions were measurable for response evaluation according to Response Evaluation Criteria In Solid Tumors version 1.1 (RECIST v.1.1) (18).

### Aeonose™ measurements

Prior to the first administration of ICIs, a breathing test was performed. A disposable mouthpiece with carbon filters was used to prevent contamination with environmental VOCs. Patients needed to wear a nose clip during their five minutes of breathing into the device. In order to avoid a confounding factor related to the device, all breath tests were performed on the same device. All breath tests were executed by healthcare practitioners with experience in performing breathing tests. During the test, patients were asked to report the extent of experienced discomfort (fear, pain and/or dyspnea) between 0 and 10.

Exhaled breath was analyzed using the CE-certified Aeonose™ device from the eNose Company, Zutphen, the Netherlands. The Aeonose™ is an eNose device incorporating three metal-oxide sensors and records VOC patterns in exhaled breath. The VOCs in the exhaled breath provoke oxidation or reduction, called redox reaction, on the surface of three sensors that subsequently change the measured conductivity due to temperature profiles. The Aeonose™ uses thermal cycling, which generates specific and unique VOC pattern by recording the passing of this thermal cycle with each sensor. The Aeonose™ is equipped with a pump with two inlets. One inlet is connected to an active carbon filter to provide a baseline measurement free from environmental influence, while the second inlet is attached to the breathing tube. These inlets are controlled by a solenoid switching between the two different inlets, facilitating an active airflow across the sensors. The metal-oxide sensors are periodically heated in cycles of 20 seconds in 64 steps.

The records of the Aeonose™ measurements were transferred to a secure server by means of an iOS device using the AeonoseDatacollector app afterwards.

### Endpoints

Clinical benefit was the primary outcome measure and was determined according to best tumor response using RECIST v1.1 (18). Results of patients with complete response (CR), partial response (PR) and stable disease (SD) as best tumor response were classified as positive (clinical benefit from ICIs), whereas the results of patients with progressive disease (PD) were classified as negative (without clinical benefit). For the performance of eNose to predict best tumor response, sensitivity, specificity, accuracy and positive and negative predicted value were determined.

### Data analysis

**Figure 1:**
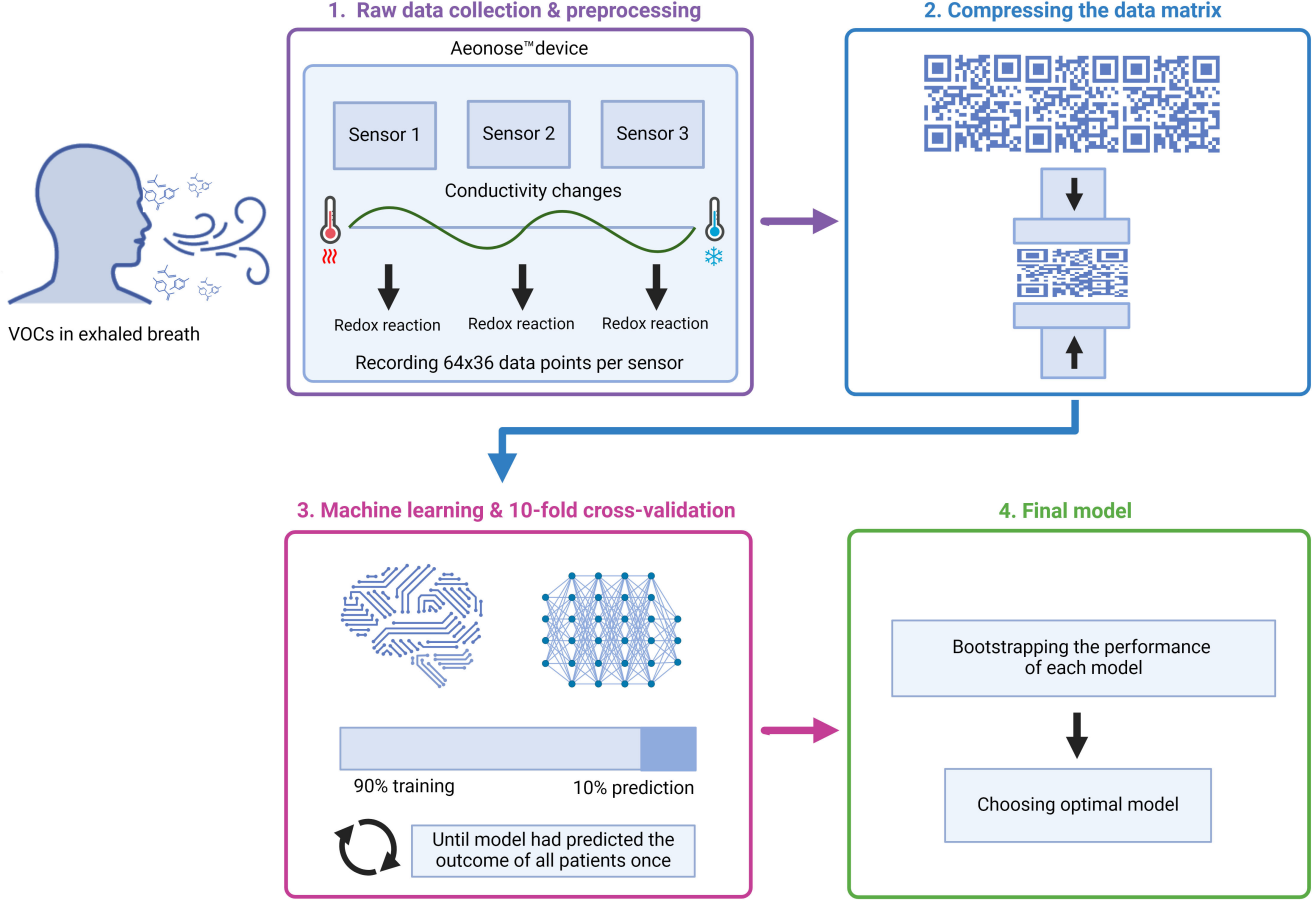
Electronic nose (Aeonose™) technology and model development.

Aeonose™ data processing and analyses have been described previously in detail (19). In summary, the procedure included preprocessing, data compression, model selection and cross validation. Preprocessing was done by normalizing the data to correct for aging of the sensors over time. The obtained data of the exhaled breath were analyzed using the Aethena software package (19). During an exhaled-breath measurement (**Figure 1**), 64 x 36 data points were recorded for each of the three sensors. For every measurement, the generated data consisted of a matrix with thousands of records. To avoid overfitting and remove redundant information and noise, the acquired data were compressed using singular value decomposition (SVD). Thereafter, since the records were too large for classification, the generated vectors were used to train a Random Forest classifier.

Machine learning was used to develop a model to identify patients with and without clinical benefit after treatment with ICIs according to the exhaled VOC patterns. The model was developed using a training set. A train-test split ratio of 80–20% was applied. To train the developed machine-learning model, 10-fold cross-validation was used where the model used 90% of the data for training and the remaining 10% of the data for prediction. The cross-validation was repeated with another 90% of the data, until the model had predicted all patients once. Using this technique ensures the data is not based on coincidences or overfitting data, but on actual differences between patients. Analyses yielded values between –1 and 1 per subject, where a value of +1 represents a perfect prediction, 0 no better than random prediction and -1 indicates total disagreement between prediction and observation.

After bootstrapping the performance of each model, the optimal model was chosen (**Figure 1**). A cut-off value for the probability of clinical benefit to ICI was determined for the training set to obtain the optimal discrimination performance, as deemed relevant for clinical practice.

### Statistical analysis

Demographic data and baseline characteristics were summarized using means and standard deviations for normally distributed continuous data, or median and interquartile ranges for non-normally distributed data. Characteristics of patients with and without clinical benefit were compared using the independent-sample t-test for continuous variables and the Fisher’s Exact Test for dichotomous variables. The potential factors that might have an influence on the VOC composition were compared between the true positive and false positive predicted outcomes (clinical benefit *versus* without clinical benefit) using the Fisher’s Exact Test.

Primary parameters of diagnostic relevance included sensitivity, specificity, accuracy, positive predictive value (PPV), negative predictive value (NPV), and the area under the curve (AUC), calculated with 95% CIs, of the receiver operating characteristics curve (ROC-curve).

Although a sufficient sample size is required for correct classification in a machine learning study, the exact sample size calculation is not reliable for such a pilot study. In a previous proof-of-concept study with an eNose, 25 patients were required in each study group (20–25). We determined the minimal sample size of patients with (n=25) and without (n=25) clinical benefit from ICIs. However, as the number of included patients increases, a more stable and robust model can be developed. Given that the expected response rate of combination therapy with ipilimumab plus nivolumab is higher compared to monotherapy pembrolizumab or nivolumab (58% *versus* 33-44%, respectively) (26–29), the final sample size was set at 75 patients to ensure a sufficient number of patients with and without clinical benefit from treatment with ICIs.

IBM SPSS Statistics version 24.0 was used to preform statistical analysis. Baseline differences were considered statistically significant with a p value < 0.05.

## Results

### Study population

**Figure 2:**
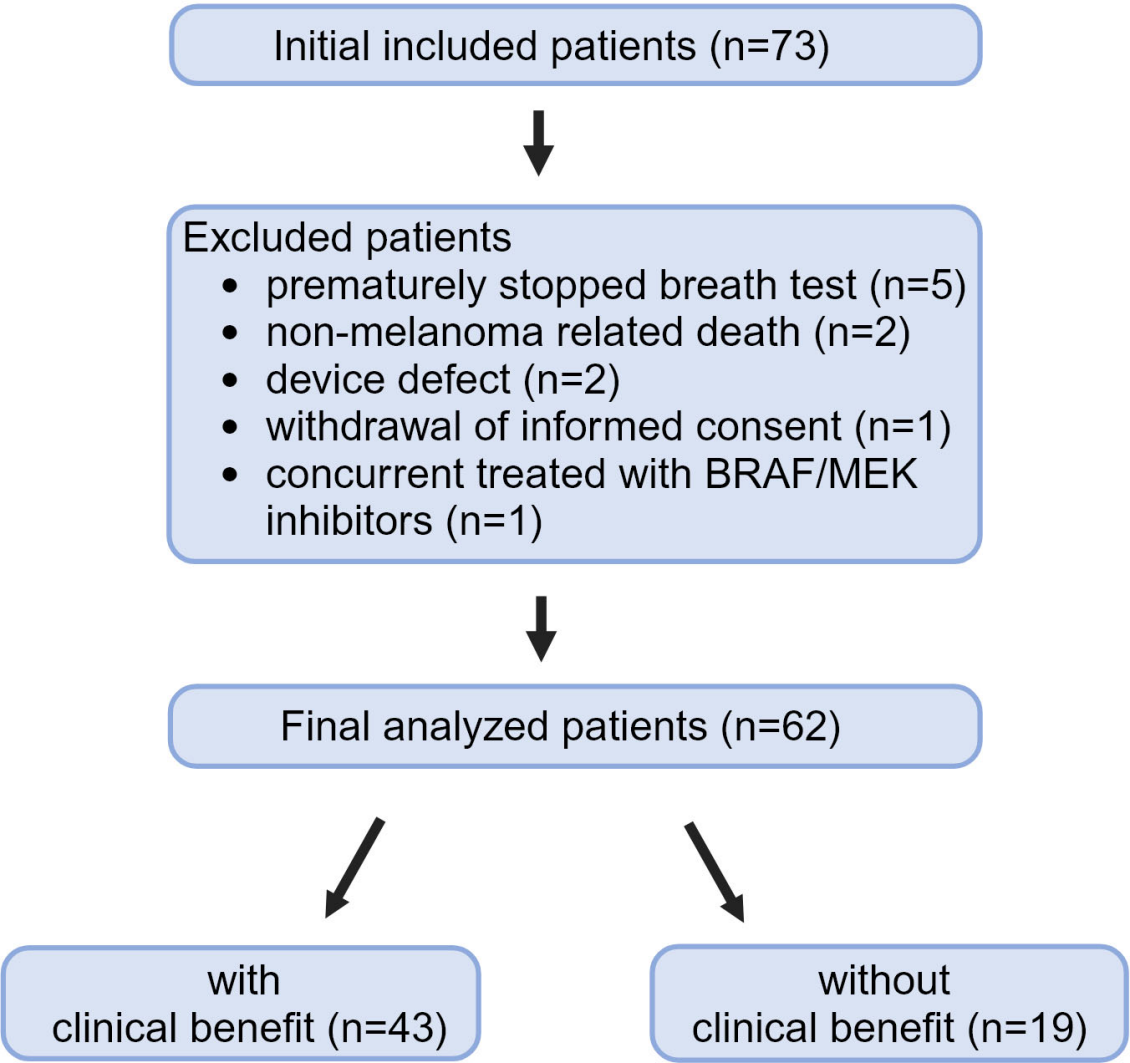
Procedure of included patients.

Between December 2020 and March 2024, 73 patients were included. The target number of 75 patients was not achieved, because the trial had to be closed prematurely. The eNose Company ceased their operations because of financial reasons in the Netherlands. Eleven (15%) of 73 patients had to be excluded from the analyses (**Figure 2**). Reasons for excluding patients from the analyses were premature discontinuation of the breathing test due to shortness of breath (n=5), non-melanoma related death before response evaluation (n=2), temporarily device defect (n=2), withdrawal of informed consent (n=1), and concurrent treatment with BRAF/MEK inhibitors before the first response evaluation (n=1). The five patients that were not able to complete the test were nonsmokers; four patients experienced shortness of breath and were not able to exhale for several seconds, one of them had very impressive lung metastasis and one patient experienced anxiety.

Finally, a total of 62 patients could be included in the analyses.

### Baseline characteristics

The total included patients were divided into two groups: patients with clinical benefit (n=43) or patients without clinical benefit (n=19). Patient characteristics are described in **Table 1**. Anti-PD1 monotherapy was prescribed in 45.2% of the included patients and 54.8% received combination therapy with ICIs. Pembrolizumab was the only anti-PD1 monotherapy administered. According to RECIST v1.1, five (8%) patients had CR, 21 (34%) patients PR, 17 (27%) patients SD and 19 (31%) patients PD, resulting in 43 (69%) patients with clinical benefit and 19 (31%) patients without clinical benefit from treatment with ICIs.

**Table 1:** Clinical characteristics of included patients.

Characteristics	(n=62)	Clinical benefit (n=43)	Without clinical benefit (n=19)	P value
Age, median in years (IQR)	72.5 (62.8-81.0)	70.0 (62.0-80.0)	73.0 (63.0-83.0)	0.745
Male, n (%)	32 (51.6)	25 (58.1)	7 (36.8)	0.434
WHO status, n (%)				0.009*
0	42 (67.7)	30 (69.8)	12 (63.2)	
1	18 (29.0)	13 (30.2)	5 (26.3)	
2	2 (3.2)	0 (0.0)	2 (10.5)	
Increased LDH^a^, n (%)	21 (33.9)	12 (27.9)	9 (47.4)	0.042*
Brain metastasis^b^, n (%)	18 (29.0)	13 (30.2)	5 (26.3)	0.902
ICI treatment, n (%)				0.002*
Pembrolizumab	28 (45.2)	23 (82.1)	5 (17.9)	
Ipilimumab plus nivolumab	34 (54.8)	20 (58.8)	14 (41.2)	

LDH, lactate dehydrogenase.

a >ULN, >250 U/L.

b Two subjects with missing data.

*significant.

The median age of patients was 72.5 years (IQR 62.8-81.0) and was not different between patients with and without clinical benefit. As compared to patients with clinical benefit, patients without clinical benefit had more frequently a significant poorer performance status (p=0.009), an elevated LDH level [> upper limit of normal (ULN), >250 U/L; p=0.042], and were more frequently treated with the combination of ipilimumab plus nivolumab (p=0.002).

### Tolerability of the test-procedure

After the breathing test using the eNose, patients who completed the test were asked to indicate the extent of discomfort (fear, pain and/or dyspnea) between 0 and 10 (0 indicating no discomfort and 10 indicating maximal discomfort) they experienced during the test. The median score was 2 (IQR 0-3). Despite the fact that they were deemed capable of performing the breathing test beforehand, five patients could not complete the test due to shortness of breath and had to be excluded from the analysis.

### Characteristics of potential factors that may affect VOC composition

**Figure 3:**
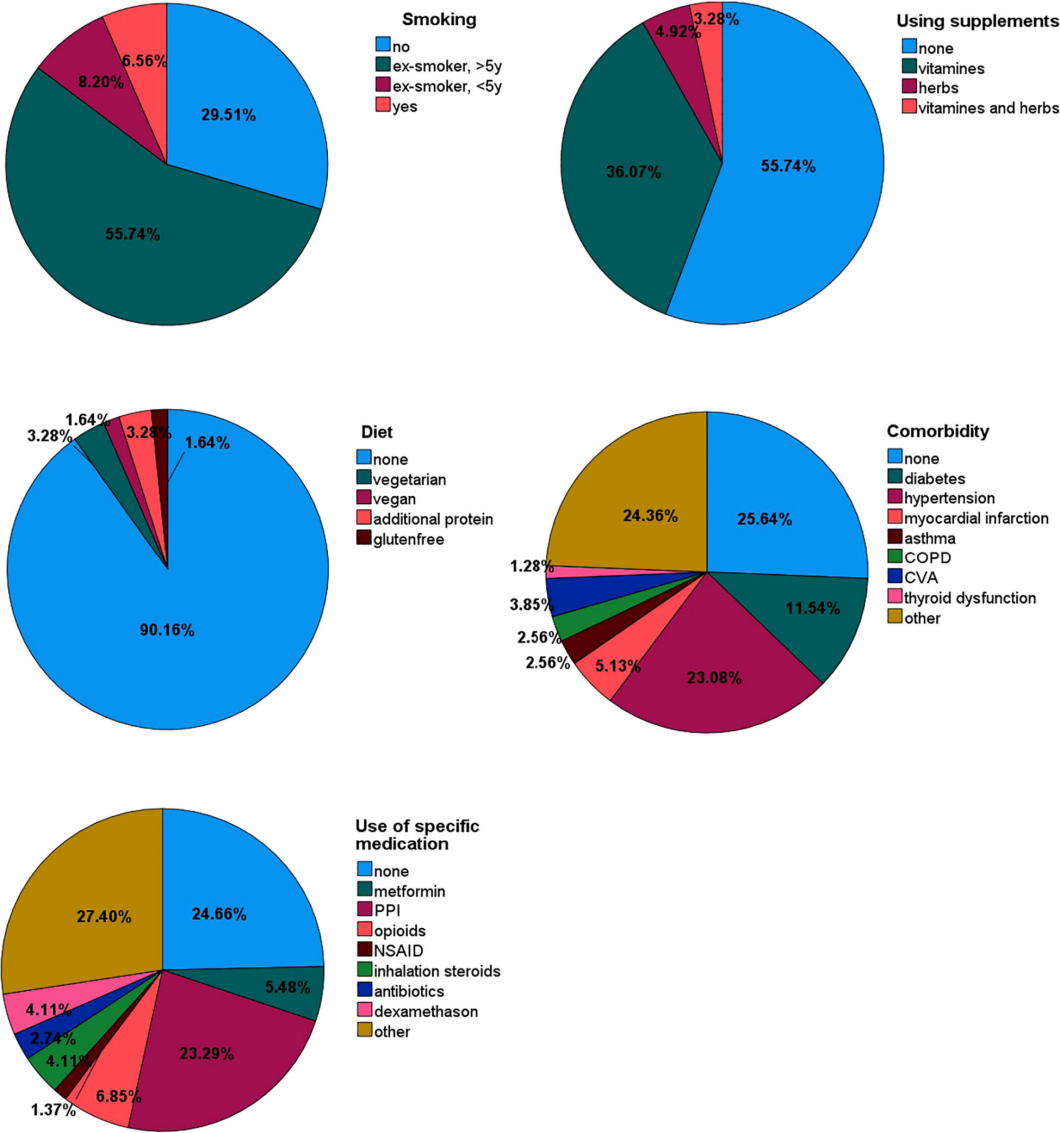
Pie charts of potential factors that may affect VOC composition, such as smoking status, supplements, diet, comorbidity and use of specific medication.

The endogenous and exogenous patient-related factors that could potentially affect VOC composition are reported in **Table 2**. The smoking status, use of supplements, diet, comorbidities and use of specific medication that could potentially affect VOC composition are reported in more detail in **Figure 3**.

**Table 2:** Characteristics of potential influencing factors of VOC composition.

Characteristics	Total (n=62)
Endogenous factors	
BMI, mean ± SD, kg/m2	26.02 ± 3.90
Specific comorbidities^a^	30 (48)
Infection	3 (5)
Exogenous factors	
Smoking^a^	4 (7)
Alcohol use in general^a^	30 (49)
Alcohol use within last 24hrs^a^	16 (26)
Specific diet^a^	6 (10)
Last meal <3hrs	34 (55)
Use of supplements^a^	27 (44)
Specific medication^b,c^	25 (32)

BMI, Body Mass Index.

*^a^* hypertension, diabetes mellitus, myocardial infarction, heart failure, asthma, chronic obstructive pulmonary disease (COPD), kidney failure, thyroid dysfunction, dementia, Parkinson’s disease, cerebrovascular accident (CVA) or infections.

*^b^* metformin, PPI, opioids, NSAID, inhalation steroids, antibiotics, dexamethasone, other.

*^c^* One subject with missing data.

### Model performance

**Figure 4:**
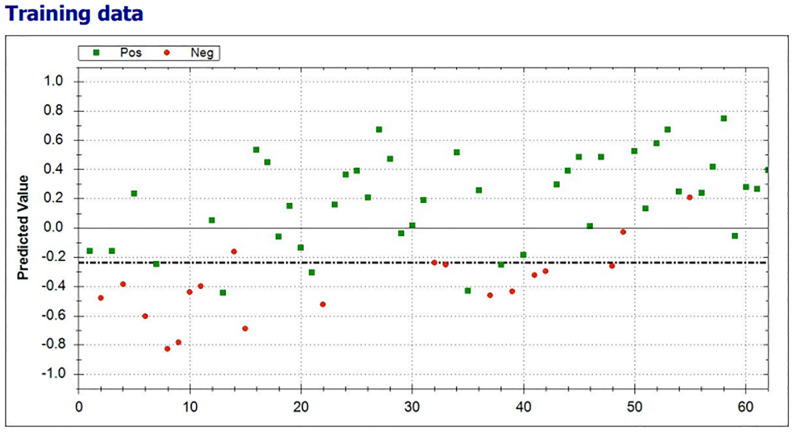
Scatterplot of the individual predictive values of the breath test with clinical benefit (green) and no clinical benefit from ICI (red) test results. The threshold was set on -0.24 (dotted line). All breath tests with a predictive value of ≥-0.24 were classified as positive.

The scatter plot of the individual predictive values of the breath test are shown in **Figure 4**. Using machine learning, the threshold was set on -0.24. All breath tests with a predictive value of ≥-0.24 were classified as positive (clinical benefit), and those with a predictive value of <-0.24 were classified as negative (without clinical benefit).

**Figure 5:**
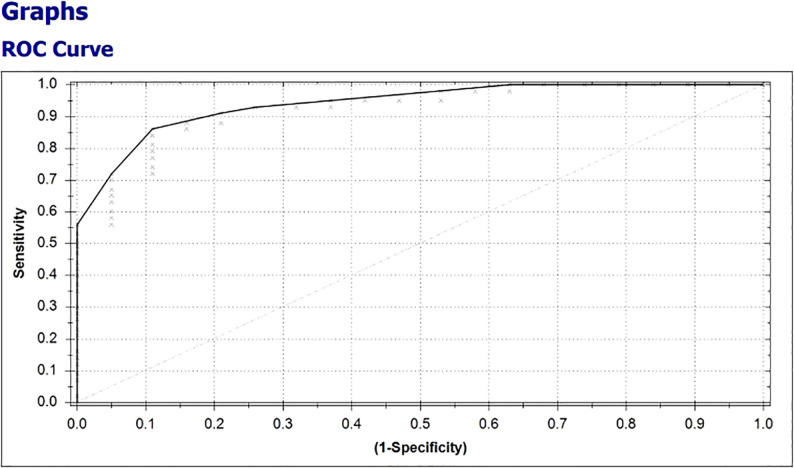
Receiver operating characteristic (ROC)-curve, representing the diagnostic value of the eNose to discriminate between breath samples from patients with and without clinical benefit from treatment with ICIs for metastatic melanoma.

In 38 of 43 (88.4%) patients with clinical benefit, the results of the eNose were true positive, whereas the results of the eNose were true negative in 15 of 19 (79%) patients without clinical benefit from treatment with ICIs. A total of four patients had a false positive test result and five patients had a false negative test result, ensuing in a positive predictive value of 0.90 (0.76-0.97) and a negative predictive value of 0.75 (0.51-0.90), respectively. To predict clinical benefit to ICIs, the eNose had a sensitivity of 88%, specificity of 79%, and accuracy of 85%. The Matthews Correlation Coefficient was 0.66 and the area under the curve (AUC) was 0.93 with the threshold of -0.24 (**Figure 5**).

To compare the erroneously predicted (n=9) and correctly predicted test results (n=53), we simplified all potential factors that might affect the VOC composition into deviant or not deviant (e.g. use of any specific medication). Both groups showed a median of two potential factors that might affect the VOC composition and there were no significant differences between both groups (p value = 0.650). No clear explanation was found for the falsely predicted test results.

## Discussion

In this prospective feasibility study, eNose was used to assess the breath pattern of exhaled breath in patients with irresectable and metastatic melanoma prior to the start of ICIs. The eNose showed a distinct breath pattern between patients with and without clinical benefit from ICIs. To predict patients who do not benefit from first-line treatment with ICIs, breath pattern analysis using the eNose resulted in a sensitivity of 88%, specificity of 79%, and accuracy of 85%.

Although lifestyle factors are known to affect fecal VOCs, as measured by an eNose (17), the effects of lifestyle factors in breath VOCs have not been investigated. Therefore, in the current study, a number of endogenous and exogenous patient-related factors were collected. Most patients had some of these factors present, but the presence of specific patient-related factors did not explain the false positive and false negative test results. These findings are confirmed by another study which showed that breath patterns that are associated with tumor response are not influenced by baseline characteristics and lifestyle of patients (15). Besides, the patient population in this study is a good representation of the real-world population and increases external validity.

Different eNoses have been developed and investigated to detect and evaluate treatment in several solid tumors. However, only two studies investigated eNoses in patients who were treated with ICIs. Another eNose (SpiroNose) was investigated in a prospective trial to identify patients with advanced NSCLC who had a tumor response to anti-PD-1 monotherapy after 6 weeks of follow up (15). Their study showed that the most sensitive sensor to methane and natural gas consistently had the largest contribution to the predictive performance of the developed model in patients who were treated with ICIs. To the best of our knowledge, the current study is the first to investigate an eNose for the prediction of clinical benefit from treatment with ICIs in patients with metastatic melanoma.

In patients with cancer, more than 130 studies reported changes of the VOCs, which were most frequently analyzed in exhaled breath (30). In most studies investigating exhaled breath, several compounds were detected, including alcohols, ketones, aldehydes and hydrocarbons. Analyses of exhaled breath using eNoses (e.g. Aeonose™, SpiroNose) are based on the analyses of patterns of the exhaled breath which generates signals from several sensors. Since these eNose techniques use pattern recognition, VOCs cannot be identified on a molecular level. Therefore, further research is needed to determine which specific VOCs contribute to discriminate between responders and non-responders to learn about specific metabolic pathways that are associated with response.

The biology of melanoma produces specific VOCs that may contribute to identify the breath pattern in patients with metastatic melanoma. Here, we discuss two potential sources of specific VOCs production, i.e. an increased lactate production and melanoma specific VOCs. First, in metastatic melanoma, a high tumor load and poor risk disease are associated with elevated levels of LDH (31). Patients with high serum LDH levels also have elevated levels of LDH isoenzymes, which drive pyruvate conversion to lactate (32). Therefore, it is conceivable that patients with elevated LDH levels produce lactate accompanies by a change in VOCs. Another contribution to an increased production of lactate is the most common change of metabolism in cancer cells, called the Warburg effect or ‘aerobic glycolysis’ (33). This change in metabolism (in normal atmospheric oxygen conditions), causes glucose to be largely fermentative with an increased production of lactate, as a potential source of the changed breath pattern.

Second, specific VOCs of melanoma have been identified in cultured human melanocyte cells and in tissue of primary melanoma (34). *In vitro*, a relative abundance of 3-methylbutyric acid (isovaleric acid) in cultured human melanoma cells was measured as compared to normal melanocytes (34). In addition, a differential expression of three VOCs (4-methyl decane, dodecane and undecane) was detected in both fresh and frozen melanoma (35).

The main limitation of this study is the small sample size since the target number of 75 included patients was not achieved due to unforeseen circumstances regarding the activities of the involved The eNose Company in the Netherlands. In addition, the number of patients with clinical benefit from ICIs was relatively high, resulting in relatively underrepresentation of non-responders in this study. However, patients from a real-world population were included in this study, which increases external validity of eNose. With chronobiology getting more attention these days, it would be interesting to know if different time schedules during the day could lead to different outcomes. Previous research showed that the breath pattern has a circadian pattern with particularly changes in lower-airway resistance across the night (36), but to the best of our knowledge the impact of chronobiology on the exhaled breath pattern of volatile organic compounds has not (yet) been investigated. In the current study, the breath test was performed during day time, prior to the first administration of the ICIs. We cannot exclude that preforming the exhaled breath test at different times of the day could have affected the results, potentially leading to false positives and/or false negatives results.

The current practice in the Netherlands is that patients with unfavorable prognostic factors, such as the presence of brain metastases and elevated LDH levels, are mainly treated with combination therapy of ipilimumab plus nivolumab, whereas patients without these unfavorable prognostic factors are treated with anti-PD1 monotherapy (26). The clinical benefit rate of patients who were treated with anti-PD1 monotherapy in this study was noticeably high (82%) as compared to the literature, where a clinical benefit rate of 54.1% was reported in patients who were treated with nivolumab in a randomized clinical trial (2). On the other hand, our clinical benefit rate was lower in patients who were treated with ipilimumab plus nivolumab compared to the best overall response rate in the literature (58.8% *versus* 70.4%) (2). This is probably explained by an effective selection of patients for treatment with anti-PD1 monotherapy. Another limitation was the absence of an external validation cohort, but this was addressed using cross-validation.

This feasibility study showed differences in the exhaled VOC patterns in patients with metastatic melanoma with and without clinical benefit from treatment with ICIs. The eNose is a non-invasive, easy-to-use, fast, portable and a relatively inexpensive tool, that has been studied in several contexts with promising results (7). The results of the current study are encouraging for future clinical trials, but external validation is required with a preferably enlarged cohort. If further research confirms this performance for the prediction of clinical benefit from first-line treatment with ICIs in patients with metastatic melanoma, it can prevent unnecessary irAEs and reduce healthcare costs in the patients without clinical benefit. In addition, it could guide clinicians to start alternative treatment strategies in an early setting, such as targeted or adoptive cell therapy.

## Conclusion

The eNose seems to be able to identify the patients with metastatic melanoma who do not benefit from anti-PD1 based treatment strategies with ICIs. If further research validates its performance, an eNose can be used to early identify patients who need alternative, potentially more effective treatment strategies to improve their survival and prevent unnecessary irAEs.

## Data Availability

The original contributions presented in the study are included in the article/supplementary material. Further inquiries can be directed to the corresponding author.
